# Viral control is required for intravenous BCG-mediated protection against tuberculosis in simian immunodeficiency virus-infected macaques

**DOI:** 10.21203/rs.3.rs-8225191/v1

**Published:** 2025-12-10

**Authors:** Solomon Jauro, Erica C. Larson, Brendon Wahlberg, Michael C. Chao, Mark A. Rodgers, Janelle L. Gleim, Luke E. Hood, Persis S. Sunny, Mallory K. Collins, Jaime A. Tomko, H. Jacob Borish, Marshall A. Malin, Pauline Maiello, Todd Demarco, Sarah M. Fortune, Philana Ling Lin, JoAnne L. Flynn, Charles A. Scanga

**Affiliations:** 1 Department of Microbiology and Molecular Genetics, University of Pittsburgh, School of Medicine, Pittsburgh, PA, USA,; 2 Center for Vaccine Research, University of Pittsburgh, School of Medicine, Pittsburgh, PA, USA,; 3 Duke Human Vaccine Institute, Duke University School of Medicine, Durham, NC, USA,; 4 Department of Pediatrics, Children’s Hospital of Pittsburgh of the University of Pittsburgh Medical Center, University of Pittsburgh, School of Medicine, Pittsburgh, PA, USA,; 5 Ragon Institute of MGH, MIT, and Harvard, Cambridge, MA, USA,; 6 Department of Immunology and Infectious Diseases, Harvard T.H. Chan School of Public Health, Boston, MA, USA;; 7 Broad Institute of MIT and Harvard, Cambridge, MA, USA.

## Abstract

Tuberculosis (TB), caused by *Mycobacterium tuberculosis* (Mtb), is a major global health challenge, especially for people living with HIV (PLWH) not on antiretroviral therapy. We previously showed that intravenous *Bacillus Calmette-Guérin* (IV-BCG) provides robust protection in SIV+ Mauritian cynomolgus macaques (MCM). Here, we evaluate whether the immunogenicity and efficacy of IV-BCG in SIV+ MCM was impaired by eliminating BCG with drugs. SIV+ macaques were treated with an anti-BCG regimen of isoniazid, rifampicin, and ethambutol (HRE), starting either 1- or 3-weeks post-BCG vaccination. Five months post-vaccination, macaques were challenged with Mtb for 12 weeks. Protection conferred by IV-BCG was significant regardless of HRE timing. However, non-controllers, characterized by high viremia and reduced CD4^+^ T cells in the lungs, were significantly less protected than controllers. Thus, the robust efficacy of IV-BCG in SIV+ MCM is retained regardless of anti-BCG drug treatment, indicating that BCG does not need to survive long to provide protection. In contrast, SIV control was necessary for full protection induced by IV-BCG. Thus, our results support that viral control is critical for vaccine-elicited protection from TB in PLWH.

## INTRODUCTION

Tuberculosis (TB) is the leading cause of death by a single infectious disease globally^1^. *Mycobacterium tuberculosis* (Mtb), the causative agent of TB, causes over 10 million new cases of active TB and approximately 1.5 million deaths each year^1^. HIV coinfection can dramatically alter the outcome of TB, increasing mortality rates in active TB and promoting reactivation of latent TB^1^. This synergy stems from HIV-induced immunosuppression, including CD4^+^ T cell depletion and dysregulation of other immune cells, compromising host immunity to Mtb^2,3^. Despite effective treatments for TB, resistance to antibiotics continues to emerge, especially in people living with HIV (PLWH)^4^. Furthermore, antimycobacterial drugs can interact adversely with anti-retroviral drugs in HIV-Mtb coinfected patients^5,6^. These challenges underscore the importance of preventing Mtb infection in PLWH.

*Bacillus Calmette-Guérin* (BCG) is a live attenuated *M. bovis* strain and the sole licensed TB vaccine. Over 4 billion people have been vaccinated with BCG, typically delivered by intradermal (ID) injection to neonates. The efficacy of BCG in preventing pulmonary TB in adolescents and adults is highly variable and influenced by many factors^7,8^. Furthermore, HIV-infected children have a higher risk of developing disseminated BCG disease following vaccination^9^, although HIV infection is not an absolute contraindication for BCG^10^.

Recent studies in non-human primate (NHP) models of TB show that delivering BCG by routes other than ID can provide superior protection from Mtb challenge^11–13^. In particular, intravenous (IV) BCG induces vigorous T cell responses and confers robust protection, even sterilizing immunity, in rhesus macaques^11,14^. We recently reported that IV-BCG was similarly immunogenic and efficacious in SIV-infected Mauritian cynomolgus macaques (MCM)^15^. We observed enhanced T cell recruitment to airways, potent humoral immunity, and sterilizing protection from Mtb in 9 of 12 MCM despite ongoing SIV infection^15^. Notably, two of the SIV+ animals in which BCG did not prevent TB exhibited higher plasma viremia^15^, suggesting that SIV control was a crucial determinant of BCG-elicited protection. The outcome of SIV infection is heterogeneous in MCM- some animals naturally control viral replication (controllers) while some do not (non-controllers)^16^. This complexity significantly impacts immune function, with non-controllers experiencing both CD4^+^ T cell depletion and impaired immune functions including altered B cell signaling and memory formation^17^, increased type I IFNs which impair anti-mycobacteria response^18^, regulatory T cell imbalance, suppressed macrophage activation and death mediated by TNF^19,20^. The variability between controllers and non-controllers provides a unique opportunity to investigate the interplay between SIV control and BCG vaccine efficacy and to understand further how viral burden influences vaccine responses in PLWH.

In this study, we investigated whether drug treatment of BCG shortly after vaccination, which should improve safety in SIV+ macaques, would also impair protection against TB. Here, we report that the duration of live BCG exposure did not influence protection, with IV-BCG remaining superior in protection compared to ID-BCG. However, IV-BCG-induced protection is dependent on SIV control by the animals, with controllers significantly better protected by vaccination than non-controllers. Furthermore, we show that T cell responses in the airways and lungs of controllers associate strongly with protection, with improved pathological scores and significantly lower bacterial burdens than in non-controllers. Collectively, our results highlight the importance of viral control to achieve vaccine-elicited protection from TB in PLWH and provide invaluable insight for effective TB vaccine strategies for this vulnerable population.

## RESULTS

### IV-BCG protection is dependent on SIV control in SIV-infected MCM

**Figure 1:**
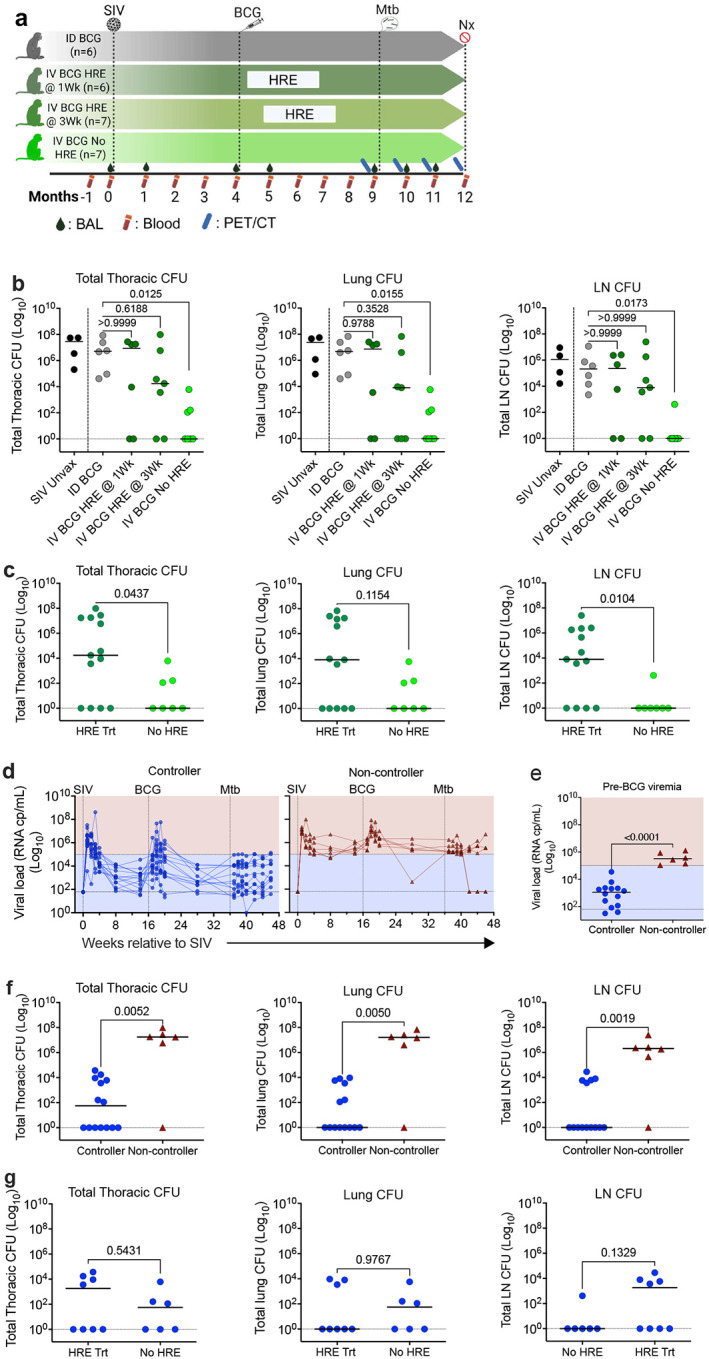
IV-BCG protection depends on SIV control, not on anti-BCG treatment. (**a**) The study outline depicts vaccination groups and the timing of SIV infection, BCG vaccination, HRE treatment, Mtb challenge, PET/CT, blood draws, BAL, and necropsy. **b**, Mtb burden in lung granulomas, lung lobes, and thoracic lymph nodes at necropsy 12 weeks post-Mtb challenge. **c**, Mtb burden, comparing HRE-treated and No-HRE groups, irrespective of SIV viremia levels. **d**, SIV levels in plasma measured longitudinally in SIV controllers (blue circles; viral load < 10^5^ cp/ml) and non-controllers (maroon triangles; viral load ≥ 10^5^ cp/ml). The limit of detection is indicated at 62 cp/ml. **e**, Plasma viremia 14 weeks post-SIV (pre-BCG) was used to declare animals as either SIV controllers (blue circles) or non-controllers (maroon triangles). Mtb burden 12 weeks post-Mtb challenge, comparing (**f**) controllers and non-controllers and (**g**) HRE-treated and No-HRE groups, excluding non-controllers. P values reported in (**b**) are from Dunn’s multiple comparison adjusted test (after Kruskal-Wallis), comparing each IV-BCG group to the ID BCG group. Mann-Whitney test was used to report the P value in panels **c**, **e**, **f**, and **g**. Each dot represents an animal, and lines represent medians.

Previously, we showed IV-BCG vaccination to be safe, immunogenic, and capable of providing excellent protection against TB in SIV-infected MCM^15^. Since BCG is a live *M. bovis* strain that can cause disseminated infection in humans, in our previous study we treated the vaccinated, SIV+ monkeys with anti-mycobacterial drugs 3 weeks after vaccination to avoid any BCG-related disease. Here, to further improve the safety of IV-BCG, we investigated whether earlier use of anti-BCG drugs post vaccination reduced the immunogenicity and protective efficacy of IV-BCG in SIV+ MCM. We administered isoniazid, rifampicin, and ethambutol (HRE) to SIV+ macaques for 8 weeks starting at either 1- or 3-weeks after IV-BCG vaccination, or we provided no anti-BCG treatment. MCM were infected with SIV (mac239 strain, 3 × 10^3^ TCID_50_, intravenously) for 4 months and then randomly assigned to 4 experimental groups: ID-BCG (5×10^5^ CFU, N=6), IV-BCG (5×10^7^ CFU) with HRE started 1 week post-vaccination (IV-BCG HRE @ 1Wk, N=6), IV-BCG (5×10^7^ CFU) with HRE started 3 weeks post-vaccination (IV-BCG HRE @ 3 Wk, N=7), and IV-BCG (5×10^7^ CFU) without HRE treatment (IV-BCG No HRE, N=7). We noted no adverse clinical effects of IV-BCG, including in the animals not receiving anti-BCG drugs. Five months post-BCG vaccination, all MCM were challenged with low-dose Mtb via bronchoscope and necropsied 3 months post-challenge (Figure. 1a, Extended Data Table 1).

At necropsy, tissue samples from lung, spleen, thoracic lymph nodes, and individual granulomas were collected, homogenized, and plated to quantify bacterial burden. Animals vaccinated with IV-BCG but not treated with HRE (IV-BCG No HRE) exhibited significantly lower total thoracic Mtb CFU compared to ID-BCG-vaccinated animals (ID BCG). However, in IV-BCG-vaccinated animals treated with HRE starting at either 1- or 3-weeks post-BCG, Mtb burden was not significantly reduced compared to ID-BCG-vaccinated SIV+ MCM (Figure 1b). Similarly, the Mtb burden in individual compartments (lung or thoracic lymph nodes) was significantly reduced in only the IV-BCG No HRE group compared to the ID-BCG group (Figure 1b). We then compared the Mtb burdens between the IV-BCG No HRE and the combined HRE-treated groups. The IV-BCG No HRE group had significantly lower Mtb bacterial burdens in both total thoracic CFU and lymph node CFU compared to the combined HRE-treated groups. However, Mtb burdens in the lungs were not significantly different between the HRE-treated and untreated groups (Figure 1c). While this suggested that treating BCG impaired protection against Mtb, we noted that there was a large range of Mtb outcomes in the IV-BCG-vaccinated HRE-treated animals with some showing complete protection and some with extensive disease (Figure 1c) and considered whether the extent of SIV control could account for this.

**Extended Data Figure 1:**
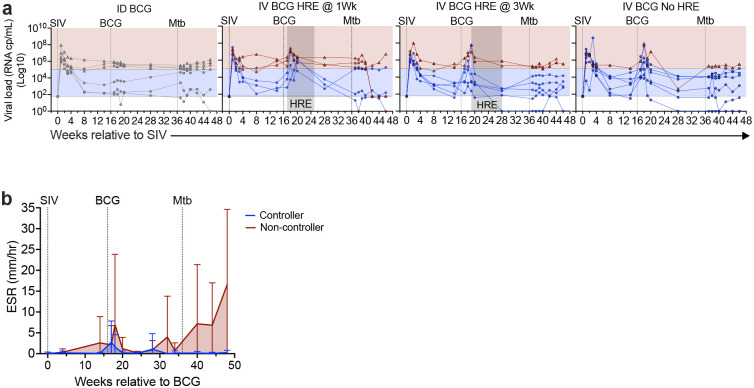
Higher SIV viremia and systemic inflammation in SIV non-controllers. **a,** Longitudinal SIV viremia by qPCR from all groups. SIV control is defined as viral load < 10^5^ cp/ml (Blue-shaded region); non-control as viral load ≥ 10^5^ cp/ml (red-shaded region). Limit of detection is 62 cp/ml. Controllers and non-controllers are identified 14 weeks post-SIV (pre-BCG) and are indicated by blue circles or maroon triangles, respectively. **b,** Longitudinal ESR of controllers (blue lines) and non-controllers (red lines). Lines are means and error bars represent standard deviation

We evaluated the plasma SIV levels in all macaques longitudinally and noted an initial surge in viral replication 2–3 weeks post-SIV infection, which declined to variable set points between 8–14 weeks post-SIV (Figure 1d, Extended Data Figure 1a). Animals that consistently sustained viremic levels ≥1× 10^5^ RNA copies/ml from 4–14 weeks post-SIV infection were designated as non-controllers (shown in red, Figure 1d, e), whereas those below this threshold were classified as controllers (blue, Figure 1d, e). As in our previous study^21^, IV-BCG, but not ID-BCG, induced transient burst in SIV replication 1–3 weeks after BCG vaccination, which returned to set point by 9 weeks post-BCG (Figure 1d, Extended Data Figure 1a). However, we noted that there were unequal proportions of controllers and non-controllers across treatment arms (Extended Data Figure 1a), suggesting that the lower level of protection associated with HRE treatment may instead be due to variable SIV control.

To determine whether the pre-BCG viremic set point was associated with IV-BCG efficacy, we compared Mtb challenge outcomes between vaccinated controllers and non-controllers. Non-controllers were significantly less protected against Mtb compared to controllers, exhibiting higher total thoracic CFU as well as significantly elevated bacterial loads in both lung and thoracic lymph nodes (Figure 1f, Extended Data Table 1). However, one SIV non-controller appeared to be fully protected, with no culturable Mtb recovered (Figure 1f, Extended Data Table 1). To determine whether treating BCG truly influenced protective efficacy, we excluded non-controllers and reanalyzed the data from IV-BCG-vaccinated SIV+ MCM in the No HRE group compared to the combined HRE-treated groups. We observed no significant difference in protection, defined by total thoracic bacterial burden, between vaccinated No HRE and HRE-treated groups (Figure 1g). This demonstrates that IV-BCG protects against Mtb challenge in all controllers, and treatment with anti-BCG drugs did not impair this protection. Instead, the ability of IV-BCG to protect against Mtb is primarily associated with the animal’s ability to control SIV replication. Accordingly, we will compare controllers with non-controllers for the remainder of this report.

### SIV controllers better restrict Mtb replication and dissemination.

**Figure 2:**
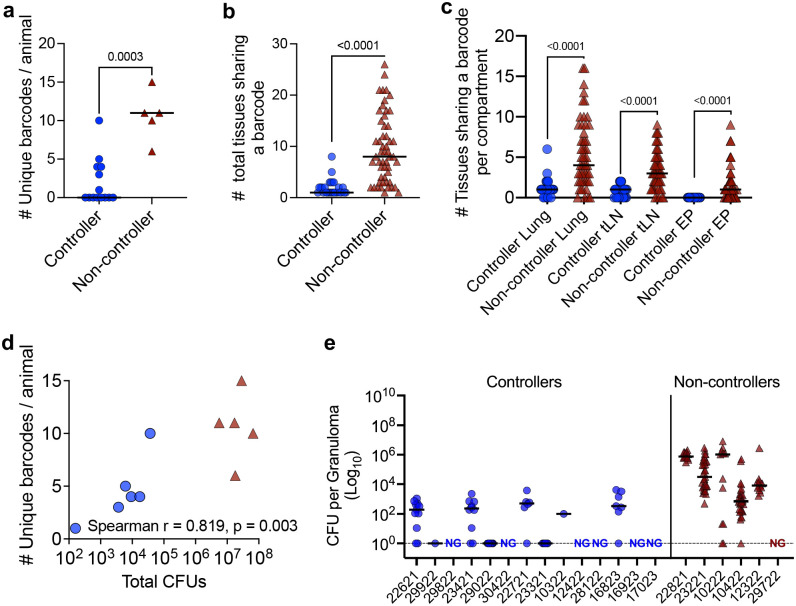
IV-BCG restricts Mtb establishment and dissemination in SIV controllers. **a**, Total number of unique Mtb barcodes isolated from each animal, reflecting the number of bacilli that established infection in controllers (blue circles) and non-controllers (maroon triangles). **b**, Total number of tissue samples sharing a given Mtb barcode, reflecting the extent of dissemination of each bacillus in controllers (blue circles) and non-controllers (maroon triangles). **c**, Number of tissue samples sharing individual barcodes in lung, thoracic lymph nodes (tLN), and extrapulmonary (EP) tissues (liver, spleen). **d**, Correlation between total number of unique barcodes and total Mtb CFU in each animal. **e**, Total number of Mtb in each granuloma from each animal is identified on x-axis, with controllers in blue circles and non-controllers in maroon triangles. NG = No Granuloma from indicated animal. **(a)** and **(d)** each dot represents an animal. (**b** and **c**) Each dot represents a barcode; lines represent medians. Mann-Whitney test was used to report the P values in (**a**) - (**c**). Spearman’s test was used to conduct correlation analysis in **d**.

The virulent Mtb challenge strain used here was molecularly barcoded, which permits tracking of bacteria in infected animals^22–25^. Animals with complete protection (culture-negative) were excluded from barcode sequencing since no Mtb was available to be sequenced. However, sterile animals were included in Figure 2a to indicate those animals that protected against establishment. Each unique barcode represents a single Mtb bacillus that established infection, and our analysis revealed more bacilli established productive infection in non-controllers than in controllers, based on the number of unique barcodes recovered in each animal (Figure 2a). Mtb dissemination from the original nidus of deposition is revealed by the number of tissue samples sharing a particular barcode. Non-controllers exhibited significantly higher Mtb dissemination than controllers (Figure 2b), with significantly increased dissemination seen in lung, thoracic lymph nodes, and extrapulmonary tissues (liver and/or spleen) in non-controllers (Figure 2c). The number of unique barcodes in controllers and non-controllers combined positively correlated with total thoracic CFU (Figure 2d).

A pre-necropsy PET/CT scan was used to identify and excise each granuloma or other pathology in the lungs^26^. Individual granulomas were homogenized and plated to quantify viable Mtb. Granulomas from vaccinated controllers had fewer Mtb compared to those from vaccinated non-controllers (Figure 2e). Thus, IV-BCG-vaccinated MCM with better SIV control were associated with fewer bacilli establishing infection upon challenge, less dissemination of Mtb, and granulomas that better restricted Mtb replication.

### IV-BCG-vaccinated SIV controllers had less pathology after Mtb challenge.

**Figure 3.**
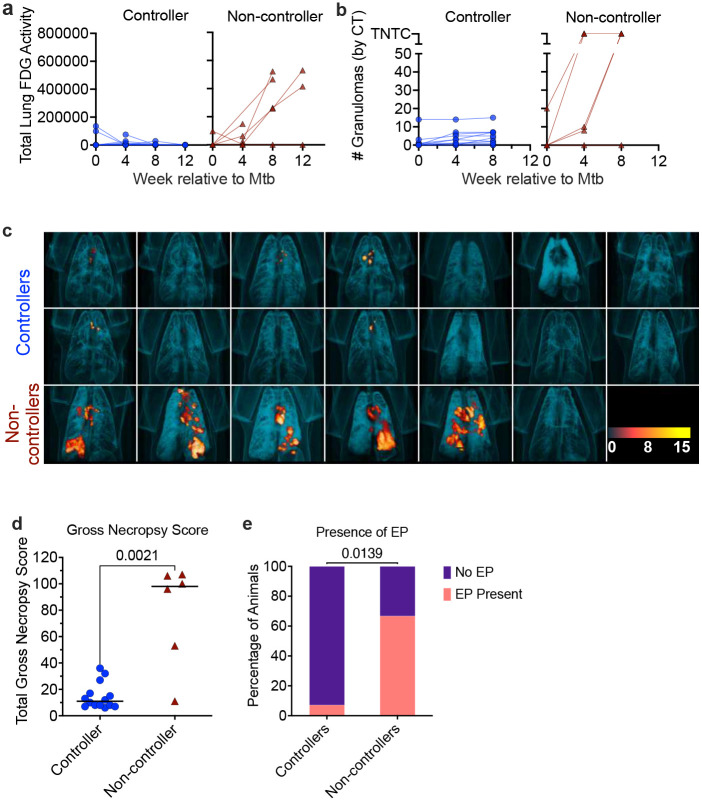
Lung inflammation, granuloma numbers, and pathology increased in SIV non-controllers. Serial measurement by PET/CT following Mtb challenge of **(a)** lung inflammation (total FDG activity) and **(b)** number of granulomas in lungs. Each line represents an animal, with controllers in blue and non-controllers in maroon. TNTC: too numerous to count. **c,** Three dimensionally-rendered PET/CT images of the thoracic cavity, obtained 12 weeks post-Mtb challenge. Controllers are shown in the top two rows, non-controllers in the bottom row. **d,** Gross pathology scores assessed at necropsy, with controllers in blue circles and non-controllers in maroon triangles. Each dot represents an animal; lines represent medians. **e,** The presence of extra-pulmonary disease (EP) was determined by gross pathology and/or growth of Mtb. Percent of controllers and non-controllers exhibiting EP is shown. Mann-Whitney test was used to report P values in (**d**) and Fisher’s exact test was used to compare prevalence of extra-pulmonary disease (**e**).

Serial PET/CT scans with ^18^F-fluorodeoxyglucose (FDG) were performed at 4, 8, and 12 weeks post-Mtb challenge to assess infection establishment and progression as well as inflammation in lungs and thoracic lymph nodes. Controllers exhibited minimal total lung FDG activity, a surrogate for inflammation, while non-controllers showed lung FDG activity steadily increasing from 4 weeks post-Mtb (Figure 3a). Notably, serial erythrocytes sedimentation rates (ESR), a non-specific measure of systemic inflammation, were consistently higher in non-controllers compared to controllers after Mtb challenge (Extended Data Figure 1b). PET/CT imaging allows us to quantify granulomas serially, where an increase in granulomas corresponds with TB progression^26,27^. Here, controllers developed fewer than 10 granulomas following Mtb challenge, except for one animal. In contrast, non-controllers displayed extensive granuloma progression and developed worse pathology (Figure 3b). Pre-necropsy PET/CT images revealed that while controllers had minimal FDG uptake in lungs, 5 of 6 (83%) SIV non-controllers had extensive FDG signal in lungs and thoracic lymph nodes (Figure 3c).

TB-related pathology was scored at necropsy using our established scoring system^26^. Controllers had significantly less pathology than non-controllers (Figure 3d). The presence of extrapulmonary pathology (EP) disease outside the thoracic cavity (e.g. spleen, liver) and whether any culturable Mtb was present in those organs^26^, were significantly lower in controllers (7.1%) compared to non-controllers (66.7%) (Figure 3e). Collectively, these results confirm that IV-BCG vaccination in controllers results in significantly better protection, with less inflammation, pathology and Mtb dissemination, compared to non-controllers.

### IV-BCG induced higher T cell influx into the airways of SIV controllers.

**Extended Data Figure 2:**
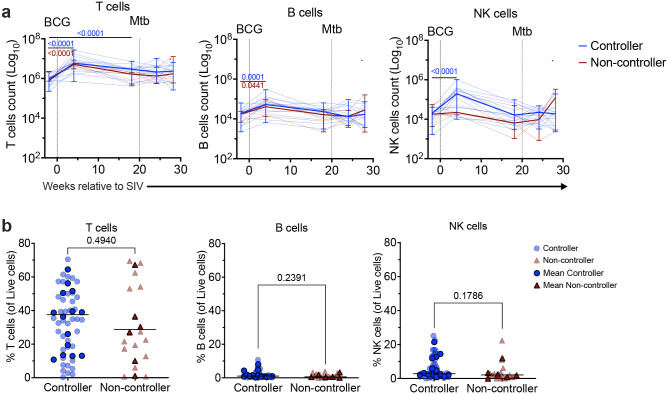
Immune cells in airways and lung tissue of SIV-infected, IV-BCG-vaccinated, Mtb-challenged MCM. **a,** The T, B, and NK cell count in airways, collected by BAL at indicated time points, indexed to time of vaccination. Thin blue and red lines represent individual controllers and non-controllers, respectively. Solid red and blue lines represent group median, and error bars represent range. Mixed-effect analysis with multiple comparisons was used. P values are from uncorrected Fisher’s LSD. Mixed-effect analysis with multiple comparisons of change over time in controllers or non-controllers are in Supplemental Data Table 2. The fixed effect (type III) are in Supplemental Data Table 3. **b,** Frequency of T, B, and NK cells in lung tissue at necropsy. Three lung lobes were collected per animal. Individual lung lobes are represented by light blue (controllers) or maroon (non-controllers); mean of all three lobes for each animal is represented by solid blue (controllers) and maroon (non-controllers) and lines represent medians. Mann-Whitney test was used to report the P values in (**b**).

**Figure 4.**
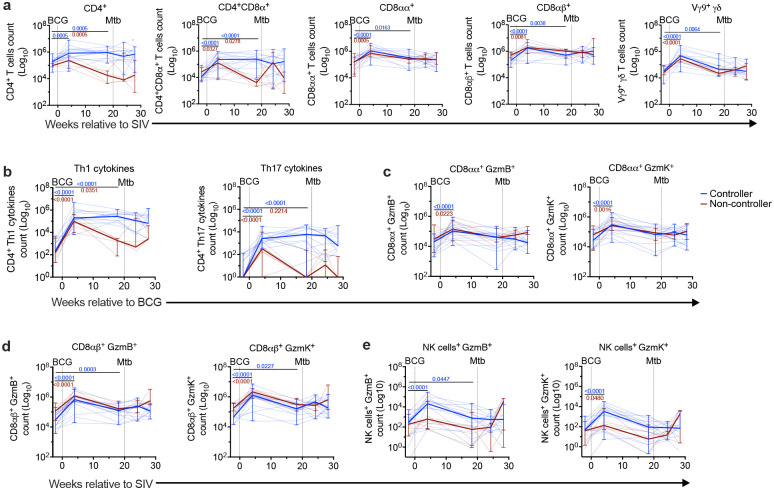
SIV controllers recruit more T cells and have more functional responses in airways. **a,** T cell subsets in airways of SIV-infected, IV-BCG-vaccinated MCM, collected by BAL at indicated time points. **b,** CD4^+^ T cells producing Th1- or Th17-associated cytokines over time. **c,** CD8*αα*^+^ T cells, **d,** CD8*αβ*^+^ T cells, and **(e)** NK cells producing GzmB^+^ or GzmK^+^ at indicated time points. The thin blue and red lines represent individual controllers and non-controllers, respectively. The thicker red and blue lines represent median of controllers and non-controllers; error bars represent range. Mixed-effect analysis with multiple comparisons was used; P values are from uncorrected Fisher’s LSD tests. Red P value indicates changes over time in non-controllers; blue P value indicates changes over time in controllers.

The protection against Mtb conferred by IV-BCG is associated with the influx of T cells and natural killer (NK) cells in the airways of both SIV-infected MCM as well as SIV-naive rhesus^11,14,15^. We used flow cytometry to evaluate the effect of viral control in SIV-infected MCM on the IV-BCG-induced influx of T, B, and NK cells in the airways. We observed a significant increase in T, B and NK, cells in controllers and only B cells in non-controllers 4 weeks after BCG, but by 18 weeks post-BCG, only controllers retained significantly more T and B cells (Extended Data Figure 2a). Further analysis of T cell subsets showed a significant increase in CD4^+^ and CD4^+^CD8α^+^ T cells 4 weeks post-BCG in the airways of controllers, which remained elevated until necropsy compared to the pre-BCG time point (Figure 4a). Conversely, in non-controllers, CD4^+^CD8α^+^ T cells significantly increased 4 weeks post-BCG, significantly decreased by 18 weeks post-BCG, and then rebounded after Mtb challenge. CD8αα^+^, CD8αβ^+^, and Vγ9^+^ γδ T cell numbers in airways increased significantly in both controllers and non-controllers, but remained significant at 18 weeks post-BCG only in controllers (Figure 4a).

Cytokines such as IFN-γ, IL-2, IL-17, and TNF are critical for protection against TB^12,28–30^. We quantified CD4^+^ T cells producing Th1 (IFN-γ, IL-2, TNF) and Th17 (IL-17) cytokines in the airways of SIV+ MCM, stratified by SIV control status, following restimulation with mycobacterial whole cell lysate (WCL). Both controllers and non-controllers showed a significant increase in CD4^+^ T cells producing Th1- and Th17-cytokines in airways 4 weeks post-BCG. However, by 18 weeks post-BCG, these responses declined to near pre-BCG level in non-controllers while remaining elevated in controllers, resulting in a significant difference between the groups at the time of Mtb challenge (Figure 4b). Following Mtb challenge, CD4^+^ T cells with Th1 or Th17 profiles remain higher in the airways of controllers (Figure 4b). The elevated cytokine production by CD4^+^ T cells in airways of controllers underscores their enhanced immunological capacity to respond to Mtb challenge compared to non-controllers.

We assessed the expression of granzyme B (GzmB) and granzyme K (GzmK) cytotoxic effectors by CD8αα^+^, CD8αβ^+^, and NK cells in the airways of controllers and non-controllers. Four weeks after BGC vaccination, CD8αα^+^ and CD8αβ^+^ T cells producing GzmB, and GzmK increased significantly in both controllers and non-controllers; however, only controllers sustained this GzmB and GzmK response (Figure 4c and d). NK cells producing GzmB increased significantly in controllers 4 weeks post-BCG and remained elevated until pre-Mtb challenge. However, both controllers and non-controllers showed a significant increase in NK cells producing GzmK in airways at 4 weeks post-BCG, which declined to pre-BCG level at 18 weeks post-BCG (Figure 4e).

### SIV controllers have robust immune responses induced by IV-BCG in lung tissue

**Figure 5.**
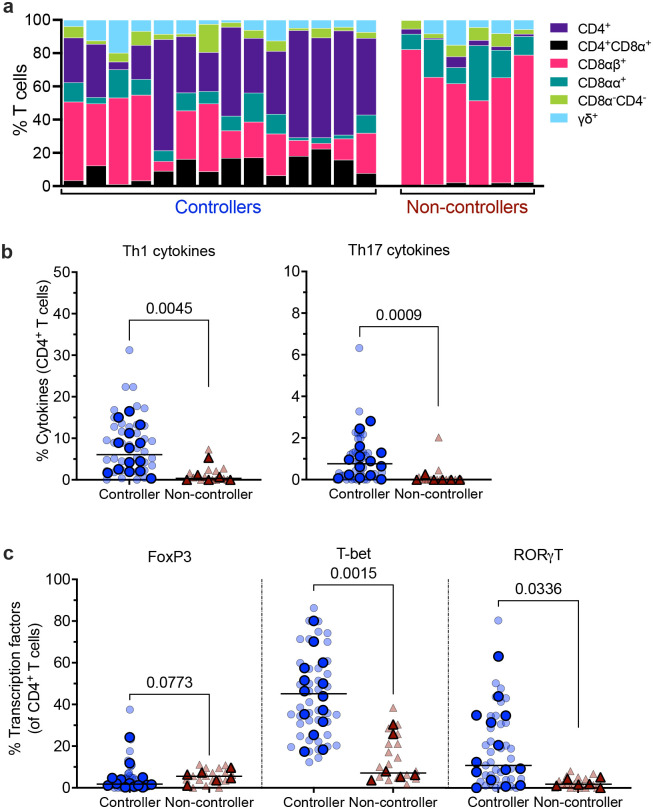
IV-BCG induces a more robust functional CD4^+^ T cell response in lungs from SIV controllers than from non-controllers. **a,** Stacked bar representation of T cell proportions in lung tissue at necropsy. **b,** Th1- and Th17-associated cytokines produced by CD4^+^ T cells in lungs. **c,** CD4^+^ T cells producing FoxP3, RORγT, and T-bet in lungs. Three lung lobes were sampled per animal; light colors are individual lung lobes and solid colors are means of all three lung lobes per animal; lines represent medians Mann-Whitney test was used to report P values in (**b**) and (**c**).

At necropsy, T cell responses were assessed in lung tissue. Both controllers and non-controllers had comparable proportions of T, B, and NK cells in the lung (Extended Data Figure 2b). However, we observed some distinct differences in T cell subsets. As expected, controllers showed significantly higher proportions of CD4^+^ and CD4^+^CD8α^+^ T cells compared to non-controllers. In contrast, non-controllers had higher proportions of CD8αα^+^ and CD8αβ^+^ T cells, with almost complete depletion of CD4^+^ T cells in their lungs (Figure 5a). We profiled Th1 and Th17 cytokines produced by CD4^+^ T cells after WCL restimulation. Frequencies of CD4^+^ T cells producing these cytokines were significantly higher in controllers than in non-controllers (Figure 5b). We evaluated transcription factors expressed by CD4^+^ T cells in lung, focusing on Foxp3, RORγT, and T-bet, which are associated with T regulatory (Treg), Th17, and Th1, respectively^31,32^. Non-controllers showed a trend towards increased frequencies of Foxp3^+^ Treg in the lungs compared to controllers. However, frequencies of CD4^+^ expressing RORγT and T-bet were significantly higher in lungs of controllers than non-controllers (Figure 5c). Our results highlight that controllers retained robust CD4^+^ T cells in lung with enhanced Th1 and Th17 responses. In contrast, non-controllers had profound CD4^+^ T cell depletion and slightly increased Tregs.

### SIV controllers show increased cytotoxic NK cells in the lung

**Figure 6.**
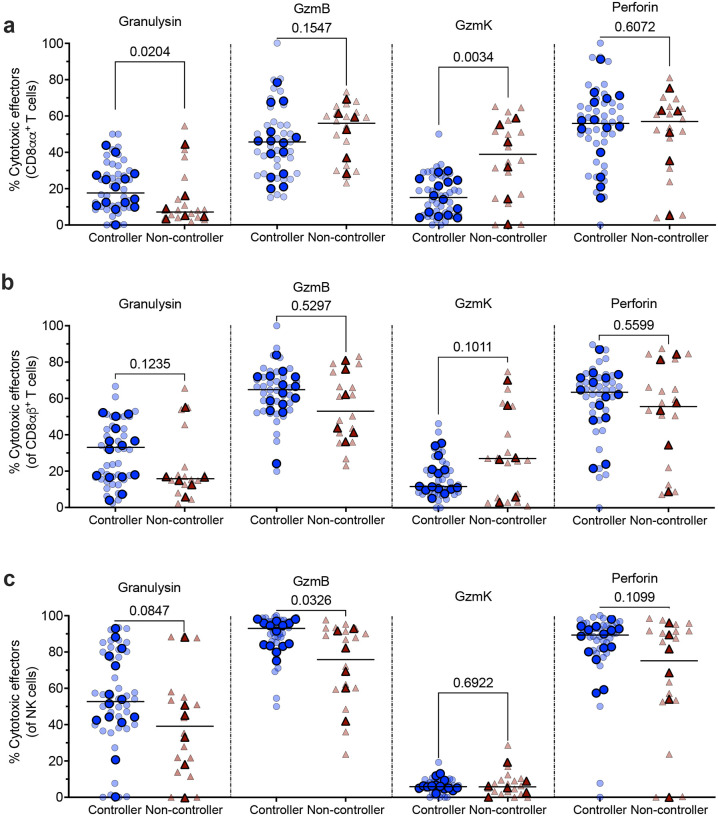
CD8*αα*^+^ T and NK cells express modestly more cytotoxic effectors in lung tissue of SIV controllers. **a,** CD8*αα* and **(b)** CD8*αβ* T cells producing granulysin, GzmB, GzmK, and perforin in lungs at necropsy. **c,** NK cells producing granulysin, GzmB, GzmK, and perforin in lungs. Three lung lobes were collected per animal; light colors are individual lung lobes and solid colors are the mean of all three lung lobes per animal; lines represent medians. Mann-Whitney test was used to report the P values.

We investigated cytotoxic effectors, namely granulysin, GzmB, GzmK, and perforin produced by CD8αα^+^ and CD8αβ^+^ T cells and NK cells in lungs of controllers and non-controllers. Cytotoxic effectors play a crucial role in host defense against Mtb^33,34^. We found that CD8αα^+^ T cells expressed significantly higher frequencies of granulysin but significantly lower frequencies of GzmK in controllers than in non-controllers. GzmB and perforin production by CD8αα^+^ T cells were similar between controllers and non-controllers (Figure 6a). Expression of granulysin, GzmB, and perforin by CD8αβ^+^ T cells was modestly higher, although not significantly different in controllers compared to non-controllers, while CD8αβ^+^ T cells producing GzmK were slightly higher in non-controllers (Figure 6b). NK cells in lungs of controllers showed a trend towards higher expression of granulysin and perforin and had significantly higher expression of GzmB production compared to non-controllers (Figure 6c). Thus, cytotoxic NK cells or other “innate-like” T cells (CD8αα^+^) in lung are associated with controllers and may contribute to better protection from Mtb following IV-BCG vaccination.

## Discussion

IV-BCG vaccination of NHP was first explored fifty years ago^35^. More recently, IV-BCG gained renewed interest when we and others showed that the IV route confers unexpected protection against Mtb in SIV-naïve macaques by inducing robust immune responses in airways and lungs^11,14,15^. We also showed that IV-BCG was immunogenic and protected 75% of macaques chronically infected with SIV against Mtb challenge, even when anti-BCG treatment was implemented 3 weeks post-vaccination to prevent BCG dissemination in case of SIV-associated immunocompromise^15^.

Here we sought to determine whether treating BCG starting 1- or 3-weeks post-vaccination was inferior in terms of IV-BCG-elicited protection compared to animals not treated with anti-BCG drugs. Using our SIV/Mtb co-infection model, we demonstrated that administering an anti-BCG regimen starting either 1- or 3-weeks post-BCG did not compromise the immunogenicity and protective efficacy of IV-BCG against Mtb challenge in SIV-infected MCM. Thus, a very short exposure to live BCG delivered IV still protects against Mtb. Notably, even without anti-BCG treatment, SIV-infected macaques did not display adverse effects of IV-BCG, nor any sign of disseminated BCG. Instead, we found that protection by IV-BCG was most robust in macaques that controlled SIV. Thus, our results demonstrate that protection conferred by IV-BCG is contingent on the ability of MCM to control SIV replication rather than on the duration of live BCG. SIV control allows IV-BCG to induce durable, robust, and functionally competent immune responses, especially Th1 and Th17 CD4^+^ T cell responses as well as cytotoxic NK cells in the airways and lungs, which collectively contribute to early protection against Mtb in IV-BCG-vaccinated SIV-infected MCM.

MCM with persistently high SIV RNA levels (≥ 10^5^ copies/ml plasma pre-BCG) were classified as non-controllers. IV-BCG vaccination of non-controllers was ineffective in protecting against Mtb. In contrast, IV-BCG vaccination of controllers resulted in protection with significantly lower Mtb burdens in lung and thoracic lymph nodes, less pathology, and little extrapulmonary dissemination. Indeed, Mtb barcode analysis showed that controllers limited Mtb establishment upon challenge and better restricted Mtb dissemination, as reported previously in SIV-naïve macaques^14,25^. The lack of protection in IV-BCG vaccinated non-controller MCM is a consequence of immune impairment by SIV, including persistently low and functionally impaired CD4^+^ T cells, and reduced cytotoxic NK cells in airways and lung tissue. These accentuate the critical role of SIV control, either naturally or with ART, in determining the efficacy of IV-BCG vaccination against Mtb, and perhaps TB vaccines using other platforms and even vaccinations for other diseases and highlight the interplay between viral and mycobacterial immunity in the setting of coinfection.

Robust T cell responses in airways are postulated to be a key immune attribute contributing to the unprecedented protection conferred by IV-BCG in SIV-naïve and SIV-infected macaques^11,15^. A correlate of protection analysis of macaques vaccinated with varying doses of IV-BCG showed that the frequency, number, and functional capacity of mycobacteria-specific CD4^+^ T cells as well as NK cell numbers in airways correlate with protection from Mtb^14^. We recently demonstrated depletion of CD4^+^ T cells or all CD8a^+^ lymphocytes prior to Mtb challenge in IV-BCG-vaccinated rhesus macaques (SIV-naive) resulted in a near-complete loss of protection. In contrast, specifically depleting only CD8ab^+^ T cells did not diminish protection by IV-BCG^25,36^. This supports that CD4^+^ T cells are critical for IV-BCG-induced protection, but also that there is a non-redundant contribution from “innate-like” CD8^+^ lymphocytes^25^. Here, we observed that IV-BCG increased T cells regardless of controller status. Nonetheless, there was a detrimental effect of SIV on CD4^+^ T cells and Th1 and Th17 cytokine production in airways of IV-BCG-vaccinated non-controllers. In contrast, controllers exhibited a markedly different CD4^+^ T cell response, characterized by robust and sustained production of Th1 and Th17 cytokines. Previous studies of SIV/Mtb co-infection and HIV infection in humans and NHP have shown that SIV or HIV infection not only ablates CD4^+^ T cells but also reduces their intralesional motility^37–39^. This implies the selective loss of highly functional CD4^+^ T cell subsets that are activated and likely produce high levels of CCR5-associated chemokines. These impairments could hinder the ability of CD4^+^ T cells to recognize and engage infected macrophages at the site of infection, a crucial step in controlling Mtb^38,40^.

Functional CD4^+^ and CD8^+^ T cells as well as NK cells were identified in the lungs of controllers. While CD8^+^ T cells were present and functional in lungs of non-controllers, CD4^+^ T cells expressing cytokines and transcription factors related to Th1 and Th17 cells were nearly ablated. Our data align with studies in both mice and NHP where a stronger T cell response in lungs correlated with killing of Mtb^11,41,42^. We reported previously that IV-BCG could induce sterilizing immunity in most animals, both SIV-naïve and SIV-infected^11,14,15^. We postulate that sterilizing immunity is established in the first 4 weeks of BCG vaccination, likely due to T resident cells in airway and luang tissue^11,14,15,21^. SIV-Mtb coinfection studies in NHP showed that depleting CD4^+^ T cells in lungs of latently Mtb-infected NHP can lead to reactivation and increased Mtb dissemination^43,44^. The lung immune responses observed in this study mirrored those in airways, suggesting a coordinated role in Mtb containment within the lung compartment.

This current study has some limitations. Our data highlighted that the protective efficacy of IV-BCG in SIV-infected MCM is dependent on the ability of the host to control SIV replication, which was supported by cellular immune response analysis using multiparameter flow cytometry. However, in-depth analysis of immune responses using more advanced techniques will elucidate more detailed mechanisms and pathways in the immune responses between SIV controllers and non-controllers. Although anti-BCG treatment did not compromise protection in this study, we did not investigate the long-term effect of anti-BCG treatment on protective immunity, which remains unknown. We also had more controllers than non-controllers in this study since animals were randomly assigned to vaccination groups. Furthermore, although our data firmly support that high viremia greatly reduces the protection afforded by IV-BCG, we did not test whether ART treatment would restore those protective effects.

In summary, our study demonstrates clearly that IV-BCG is effective even when the bacteria are alive for a short time after vaccination, supporting our previous study in SIV-naive animals using a self-killing strain of BCG^45^. However, the true determinant of IV-BCG efficacy in SIV-infected macaques is the level of viral control prior to vaccination. In particular, high viral burden was associated with depleted CD4^+^ T cells in airways and lungs as well as compromised NK cell function, two cell subsets that are likely critical in protection against TB in vaccinated animals^14,25^. Our data highlight the interplay between SIV and mycobacteria-specific immunity and provide support for ART intervention for effective vaccination against TB in PLWH.

## Online Methods

### Animals

Adult Mauritian cynomolgus macaques (MCM) *(Macaca facicularis)* were obtained from Bioculture, with animal details shown in Table S1. MCM were screened for the presence of at least one copy of the M1 MHC allele using MiSeq sequencing, conducted by the Wisconsin National Primate Center’s Genomic Service Core. MCM were housed at University of Pittsburgh. All protocols and procedures were approved by the University of Pittsburgh Institutional Animal Care and Use Committee (IACUC), which follows the standards outlined in the Guideline for Care and the Use of Laboratory Animals, the Animal Welfare Act, and the Weatherall report^46^. The University of Pittsburgh is accredited by the Association for the Assessment and Accreditation of Laboratory Animal Care International (AAALAC; accreditation number 000496).

Animal welfare was assessed twice daily, focusing on physical health indicators such as weight, appetite, and activity levels. Clinical signs of TB, such as coughing, tachypnea, dyspnea, or weight loss, were evaluated after Mtb challenge. Additionally, serial PET/CT imaging was used to monitor TB progression. All veterinary procedures were performed under sedation with ketamine or other approved sedatives. Our veterinary staff closely monitors for any sign of pain or distress and, if present, intervenes with dietary supplementation, rehydration, and/or analgesics.

### SIV infection and plasma viremia measurement

All MCM were infected intravenously with 3,000 50% tissue culture infectious dose of SIVmac239, from Dr. Koen Van Rompay, California National Primate Research Center Davis, CA. SIV RNA copies in plasma were quantified using two-step qPCR^47^. Viral RNA was extracted from 500 μL of plasma via the QIAsymphony SP platform (Qiagen, Germany) using the Virus/Pathogen DSP midi kit and cellfree500 protocol. RNA was annealed to a *gag*-specific reverse primer (5’- CAC TAG GTG TCT CTG CAC TAT CTG TTT TG −3’) and reverse-transcribed to cDNA with SuperScript^™^ III Reverse Transcriptase and RNAse Out (ThermoFisher). The cDNA was treated with RNAse H and amplified using a custom TaqMan^™^ Master Mix (ThermoFisher) with primers and a probe targeting SIVmac239 *gag*. qPCR was performed on a StepOnePlus^™^ or QuantStudio 3 system (ThermoFisher). SIV *gag* RNA copies were quantified using cycle data and a serial dilution of a SIV *gag* RNA transcript. RNA copies per ml (cp/ml) were calculated by adjusting for dilution, with a limit of quantification of approximately 62 cp/ml.

### BCG vaccination and anti-BCG treatment

MCM were randomly allocated into two vaccination groups: ID BCG (n=6) and IV-BCG (n=20). Animals were vaccinated with BCG Danish strain 1331, which was expanded at Colorado State University^48^. BCG stocks, containing approximately 3 × 10^8^ CFU/ml were stored at −80 °C. Prior to vaccination, a vial was thawed and diluted with PBS containing 0.05% Tween 80 (Sigma-Aldrich) to prevent clumping. For ID vaccination, 5 × 10^5^ CFU BCG in 1 ml PBS was injected into the dermis of the upper arm. For IV vaccination, 5 × 10^7^ CFU BCG in 1 ml PBS was administered via the saphenous vein, as done previously^15,47^.

The IV-BCG group was further divided into 3 subgroups based on the time anti-BCG treatment (HRE) was initiated (Figure 1a, Extended Data Table 1): HRE starting 1 week post vaccination (IV-BCG HRE @ 1Wk), HRE starting 3 week post vaccination (IV-BCG HRE @ 3Wk), or no HRE treatment (IV-BCG No HRE). The HRE regimen consisted of isoniazid (Teva Pharmaceuticals; 15 mg/kg), rifampicin (Darmerica; 20 mg/kg), and ethambutol (Lupin Pharmaceuticals; 55 mg/kg) and was administered orally once daily for 8 weeks.

### BAL sampling and processing

BAL was performed using a pediatric bronchoscope to instill and recover of 4 ×10 ml aliquots of warmed PBS. The collected cells are pelleted, and BAL fluid (BALF) was cryopreserved. The cells were resuspended in ELISpot media (RPMI 1640, 10% heat-inactivated human albumin, 1% L-glutamine, and 1% HEPES), counted, and distributed for flow-cytometric staining.

### Mtb challenge.

Five months after BCG vaccination, MCM were challenged by bronchoscopic instillation of 2–19 CFU of molecularly-barcoded virulent Mtb Erdman strain, as previously described^26^.

### Clinical and microbiological monitoring

Following vaccination, blood was cultured 2 and 4 weeks post-vaccination, and BAL fluid was cultured 4 weeks post-vaccination, to screen for viable BCG. After Mtb challenge, animals were closely monitored for signs of TB, including coughing, weight loss, tachypnea, and dyspnea. BAL samples and gastric aspirates were analyzed monthly post-challenge to detect any culturable Mtb. Blood samples collected at regular intervals were used to monitor the erythrocyte sedimentation rate (ESR)^26^.

### Multiparameter flow cytometry

BAL cells and cells from tissues obtained at necropsy were stained and analyzed using flow cytometry. Approximately 1 × 10^6^ cells/well were seeded in ELISpot media in a 96-well plate and stimulated for 6 hours at 37°C with either PDBu and ionomycin (P&I) or mycobacterial H37Rv whole cell lysate (WCL, 20 μl/ml; BEI resources). After 2 hours of stimulation, Golgi Plug (8 μg/ml, BD Biosciences, Cat. #555029) was added, incubated for 4 hours, and washed twice with PBS. To determine viability, cells were stained with Live/Dead dye (Zombie NIR^™^ Fixable Viability Kit; BioLegend, Cat. #423105) and incubated for 10 minutes at room temperature, followed by two PBS washes. Then, 50 μl/well of surface stain antibodies cocktail (Supplementary Data 1–4), enhanced with BD Horizon^™^ Brilliant Stain buffer (BD Biosciences, Cat. #566349), were added for 20 minutes at room temperature. Cells were permeabilized for intracellular staining (ICS) using Cytofix/Cytoperm^™^ (BD Biosciences, Cat. #554722) for 10 minutes at room temperature, followed by two washes with 1x Perm wash (BD Biosciences, Cat. #554723). For intranuclear staining (INS) of transcription factors, 1x True-Nuclear^™^ (Biolegend, Cat. #424401) was added for 45 minutes, then washed twice with True-Nuclear^™^ 1x Fix Perm wash buffer (BioLegend, Cat. #424401). Then, 50μl/well of either ICS or INS antibody cocktail was added for 20 or 30 minutes, respectively (Supplementary Data 2 and 4). Stained cells were washed twice and resuspended in 150 μl FACS buffer. A 5-color (16UV-16V-14B-10YG-8R) BD Cytek Aurora was used to run the samples and FlowJo Software for Mac OS (v10.10.0) was used for analysis. Gating strategies are shown (Supplementary Data 1–4).

### PET/CT

TB progression was monitored by PET/-CT imaging at 4, 8, and 12 weeks post-Mtb challenge using radiolabeled 2-deoxy-2-(^18^F) fluoro-d-glucose (FDG) and a MultiScan LFER-150 PET/CT scanner (Mediso Medical Imaging System), as previously described^49,50^. Images were analyzed using OsiriX MD software (v12.5.2, Pixmeo, Geneva, Switzerland) to track individual granulomas, thoracic lymph nodes, and lung inflammation over time (measured by FDG activity in the lungs).

### Necropsy, pathology, and bacterial load

Necropsies as previously described^19,27^ were performed 10–12 weeks post-Mtb challenge except for one animal necropsied 5 weeks post-Mtb for reasons unrelated to TB. Gross pathology was quantified using a scoring system^26^ to calculate the overall TB burden in each animal. Each tissue sample was divided, with a portion for histopathology fixed in 10% neutral-buffered formalin and the remainder homogenized to a single-cell suspension as previously detailed^26^. Homogenates were plated onto 7H11 agar and incubated at 37°C with 5% CO_2_ for 3 weeks to obtain total CFUs in liver, spleen, lungs, and thoracic lymph nodes. For histopathology, formalin-fixed tissue was paraffin-embedded, sectioned, and stained with hematoxylin and eosin.

### Mtb barcode analysis

Mtb DNA was extracted from the colonies on 7H11 agar plates, as described previously^51^. Briefly, an aliquot of scraped colony was vortexed with 0.1mm zirconia-silica beads (BioSpec Products, Inc.), extracted twice with phenol-chloroform-isoamyl alcohol (25:24:1, Sigma-Aldrich), and DNA precipitated with isopropanol and 3M sodium acetate (Sigma-Aldrich). DNA was resuspended in nuclease-free water (Invitrogen). Identification of the molecular barcodes has been previously described^25^. Briefly, genomic DNA was subjected to transposon-mediated fragmentation (Nextera XT, Illumina) and whole genome sequencing performed using Illumina (150 bp, paired end sequencing). All fastq reads were passed to a custom script to detect and quantitate unique barcodes associated with the Mtb Erdman library, which contains a random 7-mer barcode. To reduce bias in genome sequencing due to variable PCR amplification, and also to lower barcode sequencing depth, singleton barcodes were removed and we considered the remainder as true barcodes in each tissue. For details of our barcode detection scripts, see: https://github.com/Fortune-Lab/Mtb-WGS-barcoding

### Statistical analysis

Normality was tested using the Shapiro-Wilk test. For non-normal data, Mann-Whitney was used to compare two groups and Kruskal-Wallis test was used to compare more than two groups. For multiple pairwise comparisons, Dunn’s adjustment was used to compare each group to the control: ID BCG. To test for a relationship between two variables, Spearman’s nonparametric measurement of association was used. Serial data was analyzed utilizing a mixed effects model analysis, with Fisher’s Least Significant Difference (LSD) p-values are reported, uncorrected for multiple comparisons. Any categorical variables were compared using Fisher’s exact test. All data analysis was performed in GraphPad Prism for macOS (Version 10.5.0).

This experiment was originally designed to compare each of the IV BCG HRE-treatment regimens (HRE at 1 week, HRE at 3 weeks, and no HRE) against ID BCG-vaccinated animals. This study was powered using bacterial burden from prior studies (total thoracic CFU, log_10_-transformed, standard deviation = 0.82) as the primary outcome variable. We calculated that 6 or 7 animals per group would be sufficient to detect a mean difference of 2-logs for a two-sided t-test with α = 0.0167 (adjusted for 3 planned comparisons). For treatment groups with 7 animals, power reached 91.9%; for treatment groups with 6 animals, power reached 88.5%.

## Extended Data

**Extended Data Table 1.** Summary of vaccine groups. SIV status, sex, and age of each MCM, as well as BCG and Mtb CFUs used for vaccinating and challenging each, respectively. Shown are weeks to BCG vaccination from SIV infection, weeks to Mtb challenge from BCG vaccination, weeks to necropsy from Mtb challenge, and challenge outcome (Mtb CFU)

Vaccination groups	NHP No.	SIV status	Sex	Age	BCG dose	Mtb	Weeks to BCG	Weeks to Mtb	Weeks to Necropsy	Gross pathology score	Total CFU
**ID BCG**	229–21	NC	M	8y9m	4.60E+05	15	15.86	20	11.14	117	8.46E+07
	235–21	C	M	7y1m	3.50E+05	7	16.86	20.14	11.14	85	5.65E+06
	294–22	C	M	8y1m	5.00E+05	17	15.86	20.14	11.71	111	2.42E+07
	295–22	NC	M	10y0m	5.00E+05	17	15.86	20.14	12	41	9.12E+04
	306–22	NC	M	9y7m	2.50E+05	2	16	20	11.86	95	4.68E+06
	172–23	C	M	5y11m	3.00E+04	19	15.57	20	12.42	30	4.11E+04
**IV BCG HRE @ 1Wk**	226–21	C	M	8y11m	5.60E+07	15	15.86	20	13.14	17	9.24E+03
	232–21	NC	M	8y9m	2.30E+07	7	16.86	20.14	10.86	106	1.72E+07
	102–22*	NC	M	4y11m	5.00E+07	13	15.71	19.86	5.29*	53	2.74E+07
	123–22	NC	M	4y6m	5.10E+07	12	16.14	20	12	100	1.79E+07
	298–22	C	M	9y2m	3.70E+07	2	16	20	13	6	0.00E+00
	299–22	C	M	11y0m	3.70E+07	2	16	20	12.14	10	0.00E+00
**IV BCG HRE @ 3Wk**	228–21	NC	M	11y0m	5.60E+07	15	15.86	20	11.14	107	9.78E+07
	234–21	C	M	8y0m	2.30E+07	7	16.86	20.14	11.14	27	3.67E+04
	104–22	NC	M	5y8m	5.00E+07	13	15.71	19.86	12	96	5.76E+06
	290–22	C	M	9y7m	4.00E+07	12	15.86	20.14	11.71	36	3.62E+03
	304–22	C	M	10y10m	3.70E+07	2	16	20	12.14	8	0.00E+00
	168–23	C	M	6y0m	2.10E+07	19	15.57	20	12.14	8	0.00E+00
	169–23	C	M	6y1m	2.10E+07	19	15.57	20	12.14	32	1.73E+04
**IV BCG No HRE**	227–21	C	M	7y9m	5.60E+07	15	15.86	20	13.14	12	6.09E+03
	233–21	C	M	8y1m	2.30E+07	7	16.86	20.14	10.86	8	1.10E+02
	103–22	C	M	5y8m	5.00E+07	13	15.71	19.86	12	15	0.00E+00
	124–22	C	M	4y9m	5.10E+07	12	16.14	20	12	7	0.00E+00
	281–22	C	M	9y10m	4.00E+07	17	15.86	20.14	12	13	1.60E+02
	297–22	NC	M	11y10m	3.70E+07	2	16	20	13	11	0.00E+00
	170–23	C	M	6y2m	2.10E+07	19	15.57	20	12.43	7	0.00E+00
**SIV+ Unvax. (Historic control)**	85–18	ND	M	10y0m	N/A	16	N/A	N/A	7.86	97	5.46E+07
	87–18	ND	M	10y7m	N/A	10	N/A	N/A	10.43	79	3.45E+06
	90–18	ND	M	10y1m	N/A	16	N/A	N/A	12.14	45	2.05E+05
	174–19	ND	M	5y7m	N/A	4	N/A	N/A	13.14		5.57E+07

## Supplementary Material

Supplementary Files

This is a list of supplementary files associated with this preprint. Click to download.

- SIVIVBCGSupplementalinformationJauro.docx

## Data Availability

All relevant data are available from the corresponding author upon reasonable request
